# Supplementary figures and images for: Comprehensive Transcriptional Profiling and Mouse Phenotyping Reveals Dispensable Role for Adipose Tissue Selective Long Noncoding RNA Gm15551
Source: Noncoding RNA. 2022 May 6;8(3):32. doi: 10.3390/ncrna8030032 (PMC9149892; doi:10.3390/ncrna8030032)

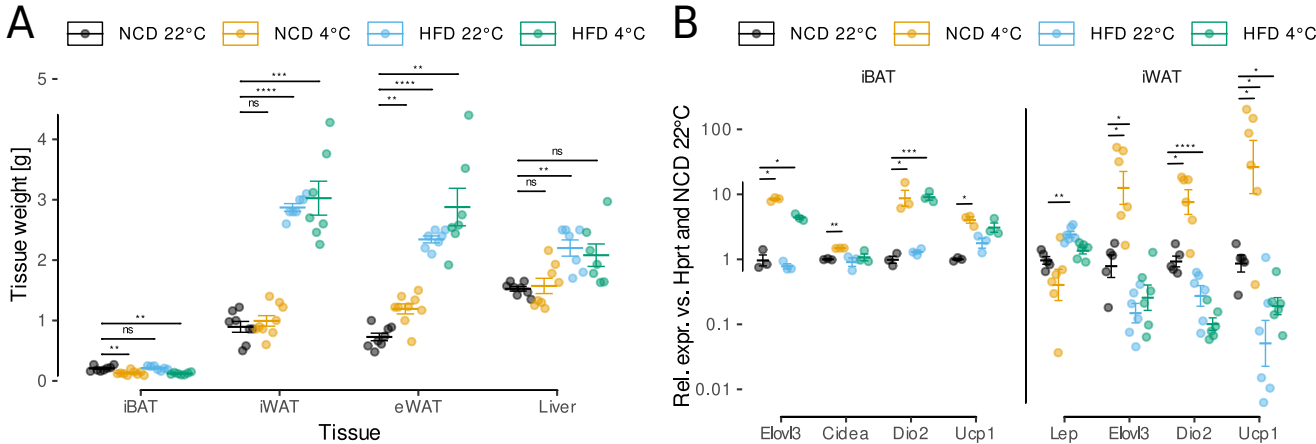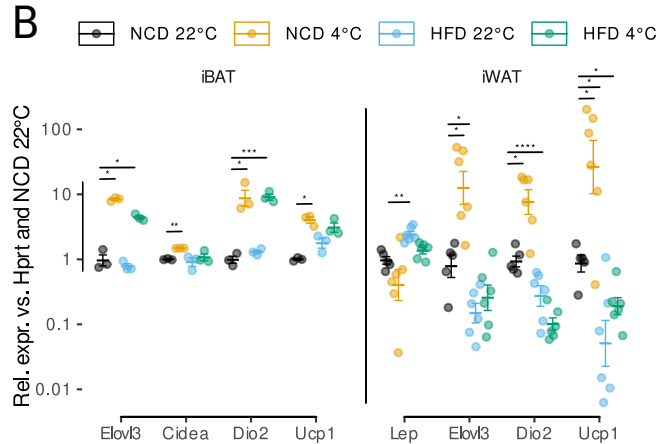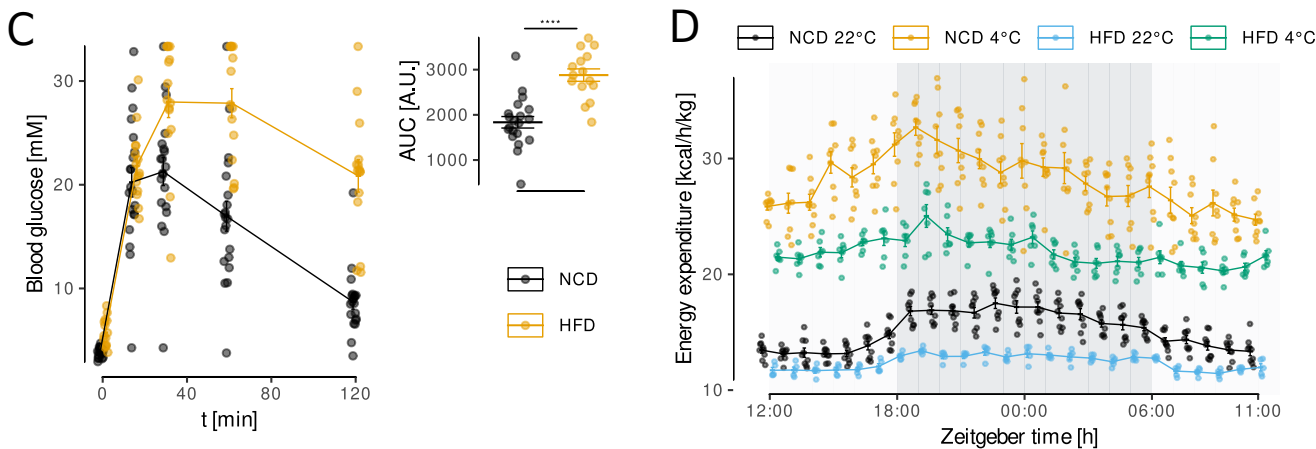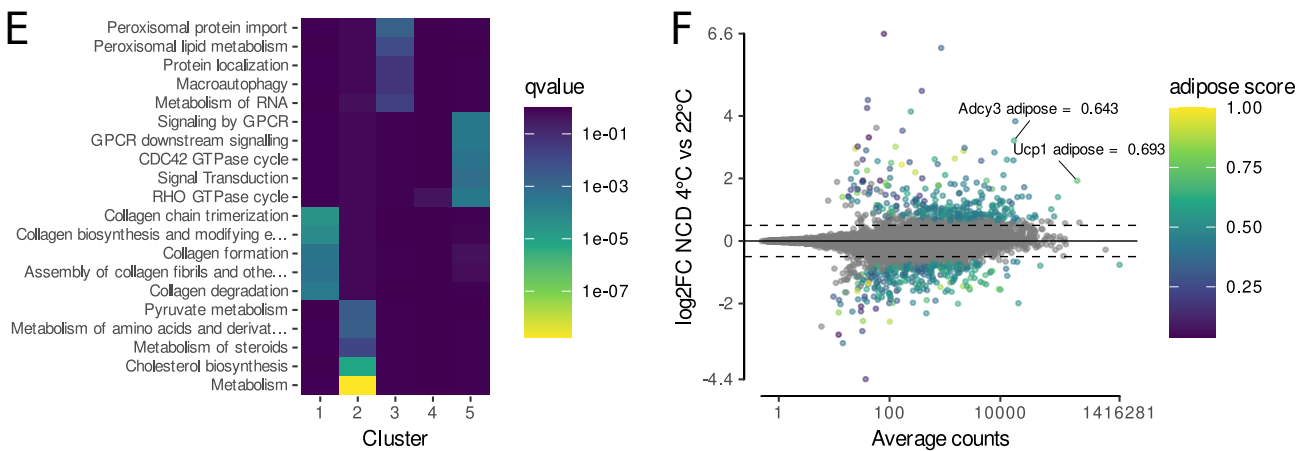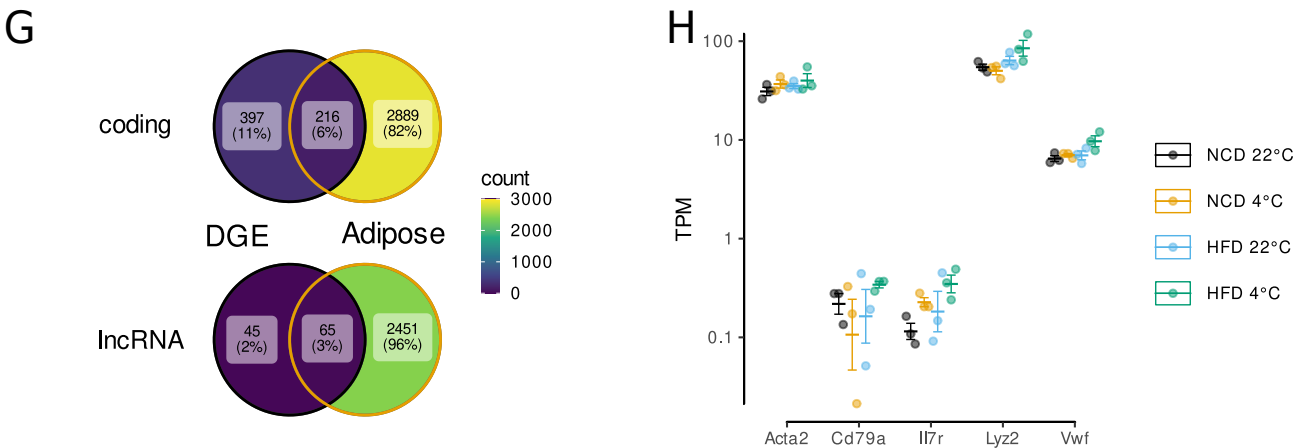

Supplement: Supplementary file 1 [file ncrna-08-00032-s001.zip › Figure S1.pdf]

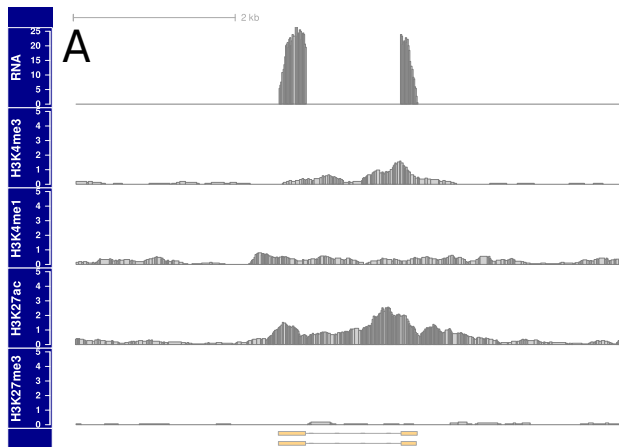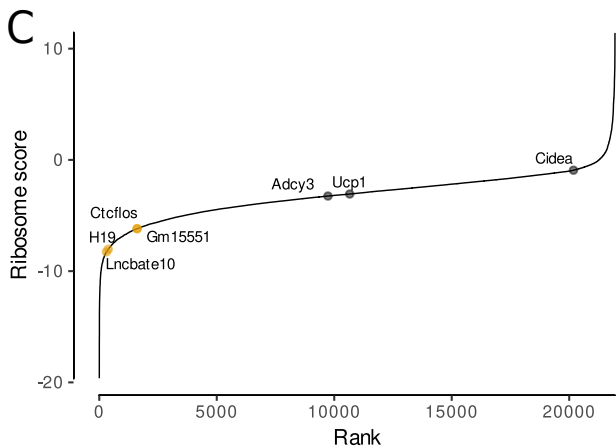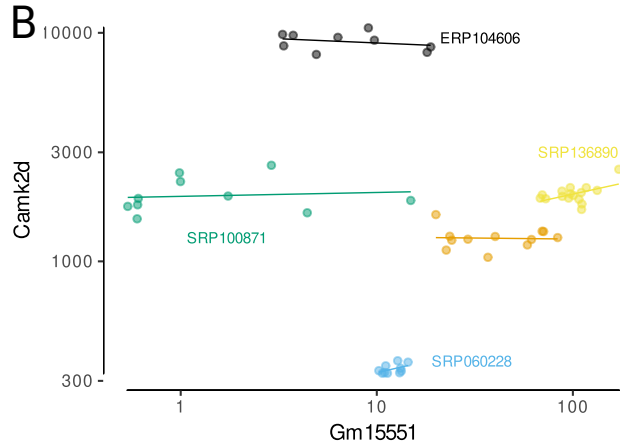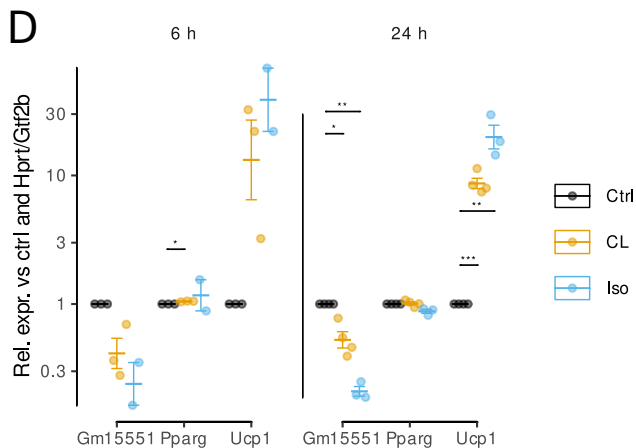

Supplement: Supplementary file 1 [file ncrna-08-00032-s001.zip › Figure S2.pdf]

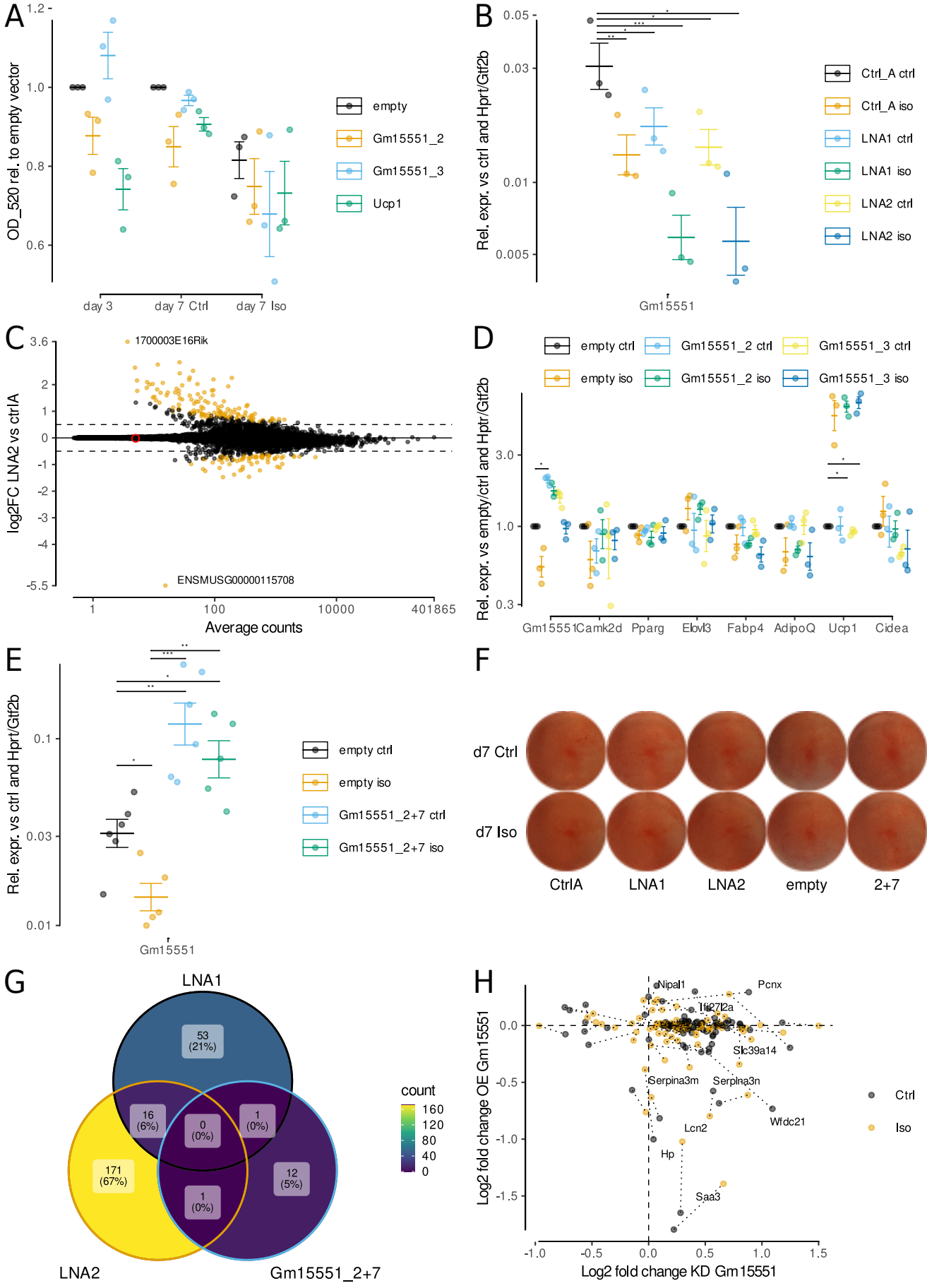

Supplement: Supplementary file 1 [file ncrna-08-00032-s001.zip › Figure S3.pdf]

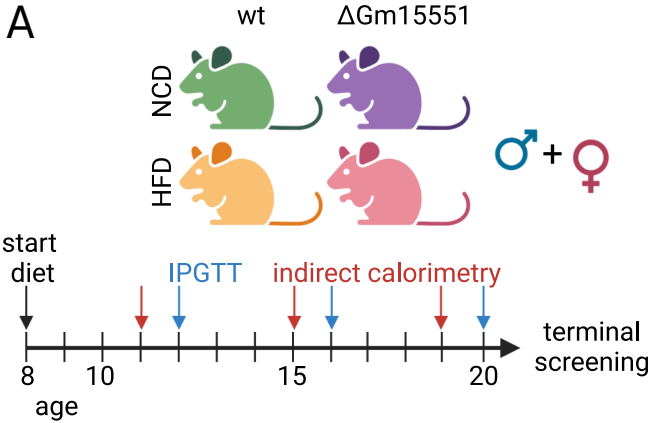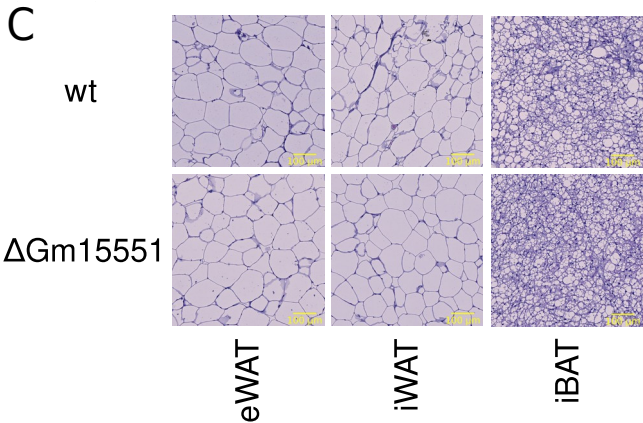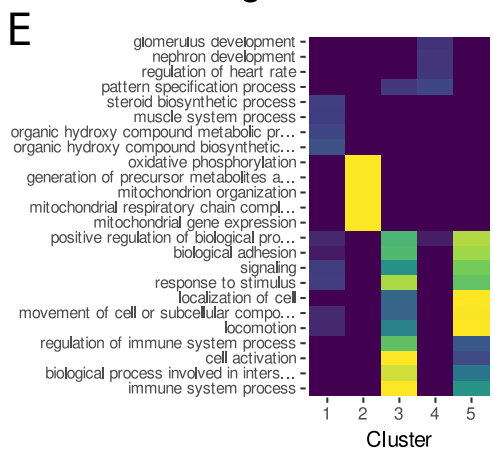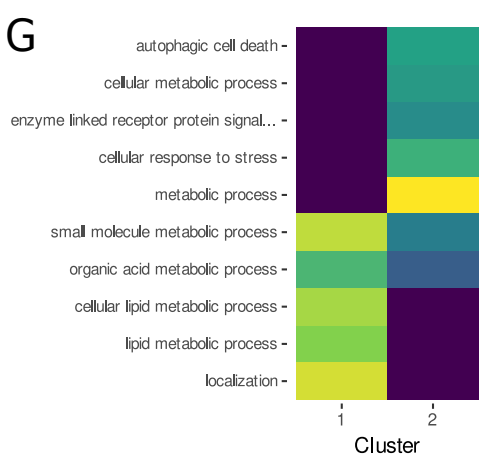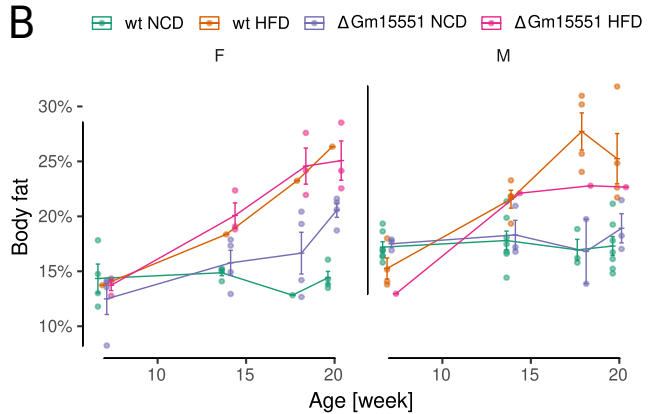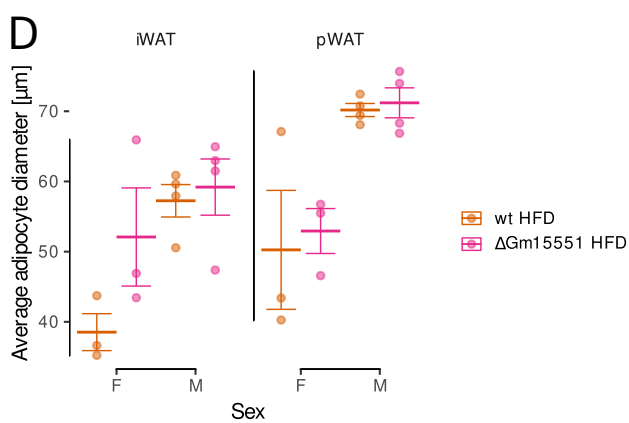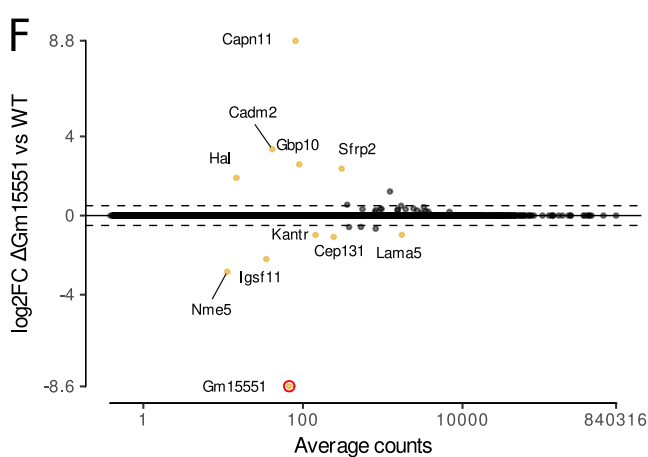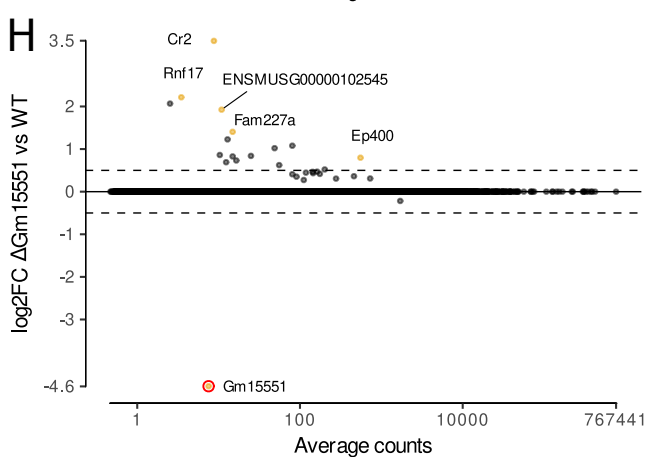

Supplement: Supplementary file 1 [file ncrna-08-00032-s001.zip › Figure S4.pdf]
